# Notes of the Week

**Published:** 1921-06-11

**Authors:** 


### NOTES OF THE WEEK.

### THE HOSPITAL CRISIS.

The wards continue closed at the London Hospital, but the prospect is brighter, and some friends have come forward with offers of help. One gentleman offered to give £5,000 if four others will give a like amount by Saturday, July 30, and his challenge has been taken up by one other. A Bazaar is also being organised by the nursing staff, and will be held at the Hospital on July 6. The series of whist-drives arranged by the *Daily Sketch* is most successful. At Holland Park 4,100 players sat down to play on Tuesday last, and it is said that almost as many failed to obtain admission.

### THE BIRTHDAY HONOURS.

Members of the medical and hospital professions do not figure very numerously in the Honours List published on June 4. In the former Knighthoods are awarded to the following:—Professor Arthur Keith, M.D., F.R.C.S., Dr. Thomas Lewis, C.B.E., M.D., F.R.C.P., Dr. Sydney Russell-Wells, M.D., and Dr. F. Conway Dwyer, M.D. Col. W. H. Willcox, C.B., C.M.G., M.D., receives a Knight Commandersip of the Order of the Indian Empire, while a Companionship of the same Order is awarded to Lieut.-Col. Benjamin H. Deare, I.M.S., and Dr. Mohendra Nath Banerjee. In the hospital world a Baronetcy is awarded to Mr. Charles A. Cain, J.P., Chairman of the Liverpool Samaritan Hospital and Governor of the Royal Southern Hospital in that city. Mr. George Mellor, J.P., receives a Knighthood. He is Chairman of the Central Fund, Lancashire, King's Military Convalescent Hospital, and has devoted himself to the development of hospital work, particularly for ex-Service men. Mr. R. W. Woolley, member of the Metropolitan Asylums Board for twenty years and its Chairman for the past six years, also receives a Knighthood.

### HOSPITALS AND COAL SUPPLY.

It is not generally known that there is a seam of coal running through the grounds of Seacroft Hospital, Leeds, and, with the sanction of the Yorkshire Miners' Association, six miners under the direction of Jack McNicholas, the well-known former footballer, are getting coal for the Killingbeck and Seacroft Hospitals from this seam, which lies in places not more than a foot below the soil. The coal yielded, though rather soft, has proved quite satisfactory as boiler-fuel. For the lighting and heating of the two hospitals a quantity between 60 and 70 tons is required weekly, and in the month during which the miners have been working in the grounds they have raised, roughly, 400 tons.

### A SUGGESTION FOR HOSPITAL SUNDAY.

We feel that it would greatly help Hospital Sunday if the action of Messrs. Haig, who paid for a page in *Punch* to benefit the Royal Waterloo Hospital, were followed by other advertisers on behalf of the hospitals of London generally. If, as in the advertisement referred to, some bonus in the shape of goods or special discount were offered to those supporting Hospital Sunday as a result of the publicity, we are sure that both charity would be helped and business stimulated. No doubt the movement would be further strengthened if the chief newspapers also gave free space to make known the aims and needs of Hospital Sunday in London.

### VACANT HOSPITAL SECRETARYSHIPS.

Following on the regretted death of Mr. Walter Alvey, a vacancy for a Secretary for Charing Cross Hospital is announced, and candidates are requested to address themselves to the Treasurer. No particulars are given as to the amount of salary or restriction, if any, in respect of the age of candidates. The Secretaryship of the Shropshire Orthopædic Hospital, Oswestry, has been filled by the appointment of Lieutenant-Colonel G. H. Thresher at a salary of £400 and a house. There were over a thousand applicants, including Admirals and Generals, and the "short list" contained, amongst others, Rear-Admirals, Commanders, Colonels, Majors and other ranks, including one curate. The vacancy at the West End Hospital for Nervous Diseases, Welbeck Street, caused by the retirement of Mr. Kirkaldy Willis, is advertised. The salary is £500 a year.

### THE HISTORY OF X-RAYS.

The Royal Photographic Society's Exhibition at Russell Square, open until the end of the month, well illustrates the short but interesting history of the *x*-ray photography. There are examples of the earliest work, such as photographs taken in 1896, and a wonderful series of plates showing progressive refinement up to the war period. At that time, of course, necessity and practice produced extraordinary developments in surgical *x*-ray work, and all its phases are amply illustrated in this exhibition. The medical profession will be particularly interested in the examples of work upon abdominal viscera.

### ANOTHER HINT TO THE METROPOLIS.

Mr. Leonard Keyser's letter to *The Times* suggesting that the London hospitals should adopt the scheme known as insurance for free hospital treatment to subscribers, which has proved successful at the Royal Hampshire Hospital, has led Dr. Collier, of the Radcliffe Infirmary, to recount the success of a somewhat similar scheme in Oxford. Like Mr. Keyser's, his letter suggests the inquiry whether similar methods cannot be employed in London. Since we have followed both these schemes from the start and described them in detail in The Hospital when they were originated, we welcome this advertisement of them in the London press. Nevertheless the fact remains that the chairmen of several London hospitals evidently believe these schemes inapplicable in the Metropolis, for they give them no countenance, and yet —with the exception of Mr. Frankau, the deputy treasurer of St. George's Hospital, whose letter we have the pleasure to publish on another page—they keep the reasons for their scepticism to themselves. But this silence is a disservice. Can it be that Lord Knutsford, for instance, who pointed out a year ago the disparity between the possible and the actual number of subscribers to the Metropolitan Saturday Fund, feels that any regular contribution scheme is the Saturday Fund's business? We can think of no better reason for his apparent indifference. Great as the difficulties are, attempts on these lines by separate hospitals in London have had some success; and this makes us believe that the scheme will eventually develop. Once again, what are the practical difficulties in its way?

### THE LANGLEY MEMORIAL PRIZE.

A prize has been instituted at the London School of Tropical Medicine in memory of Dr. W. H. Langley, C.M.G., P.M.O., of Southern Nigeria, who died in 1912. The prize is to be awarded triennially among the officers of the West African Medical Staff, and will be made in respect of the best paper written on one of the following: (*a*) Tropical medicine or surgery, (*b*) tropical hygiene and sanitation, (*c*) tropical entomology and parasitology. The first award will be in 1924, and papers should be delivered to the Secretary, Seamen's Hospital Society, Greenwich, S.E., on or before October 1 of that year.

### "EATING" GRASS.

Some attention has lately been drawn to the danger attendant upon the habit of placing grass blades in the mouth. To detail the circumstance in which the habit is prevalent is beside the point, but children and athletes provide frequent examples, and in hospital practice acute disease arising from this cause is certainly not unknown. A classical example is infection with the fungus of actinomyces; the children's game of placing certain grasses upon the tongue and allowing them to creep backwards towards the throat has frequently resulted in broncho-pneumonia. Grasses of the wayside, fresh and green though they may look, can carry a number of infections acutely dangerous to human beings.

### A PRESENTATION.

An interesting Anglo-American function took place in the Assembly Hall of the Cosmos Club, Washington, U.S.A., on May 9, when Mr. Henry S. Wellcome, well known for his generous promotion of scientific research, presented Dr. F. B. Power with a gold medal specially struck to commemorate Dr. Power's distinguished services to science during eighteen and a-half years as Director of the Wellcome Chemical Research Laboratories, London. Dr. Power's researches have been chiefly concerned with the constituents of plants, and more especially those plants used in medicine. During his directorship of the Wellcome Laboratories about 170 papers were contributed to scientific societies, and, as one of the speakers at the presentation said, Dr. Power had during that time the greatest influence in both America and Great Britain in raising the standard of our Pharmacopœias. His work is, moreover, still bearing fruit, and as an instance of this it may be mentioned that the new treatment of leprosy, which gives promise of effecting a complete cure of this terrible disease, is based on the results of a series of researches conducted by Dr. Power in London.

### THE LATE SIR JAMES HORLICK, BART.

Philanthropic and other institutions have lost a generous subscriber at the death of Sir James Horlick, Bart., on May 7, at the age of seventy-seven years. He was President of Horlick's Malted Milk Company, probably the largest firm of food manufacturers in the world, operating factories in England and the United States of America, with a business organisation extending into practically every country of the globe. Since 1895 Sir James resided at Cowley Manor, Gloucestershire, and took an active part in the public affairs of the county. He was a magistrate and deputy-lieutenant for this county, and in 1902 was High Sheriff. All forms of sport appealed to him, especially shooting, and he was a keen, good shot. He was also a well-known breeder of Shorthorn cattle and Oxford Down sheep. He was a willing and generous benefactor to many charitable and religious institutions; among his gifts were considerable sums for restoration work at Gloucester Cathedral, and he recently defrayed the cost of the reconstruction and enlargement of the Cathedral organ as a memorial to his youngest son, Major G. N. Horlick, Royal Gloucestershire Hussars, who died on active service.

### OIL COOKERS IN HOSPITALS.

At least one hospital, the Royal Hampshire County Hospital, is seriously considering the installation of oil for cooking and heating. Mr. Noel Hanbury, the chairman of the Committee, and Mr. Herbert Maslen report that a scheme is in preparation to convert the present system to the use of oil in place of other fuel. In the opinion of experts, we understand, the change is expected to be not only practical, but "a great economy." The details of the scheme are awaited with much interest. The domestic expenditure continues to increase, in spite of a reduction of thirteen in the number of patients when compared with that of the preceding year. The increase was seen in the cost of coal, gas and electricity, and especially in the cost of coal.

### HOSPITAL CONDITIONS IN VIENNA.

Sir Philip Gibbs tells a pathetic story of life in Austria after the war. In the great hospitals of Vienna, where were some of the best medical schools in the world before the war, it is almost impossible to carry on the work, owing to the dearth of supplies. In the cold weather fuel was the chief need, and many wards had to be closed down because they could not be heated at all. The position of the professional classes is no better than that of the very poor. A young Austrian doctor at a clinic said that he had lived for three years mostly on cabbage soup, with now and then potatoes for a treat, and not in all that time had he eaten meat. The clothes he wore dated from before the war, and had already been turned once, new clothes being out of the question.

